# Opposing Morphogenetic Defects on Dendrites and Mossy Fibers of Dentate Granular Neurons in CRMP3-Deficient Mice

**DOI:** 10.3390/brainsci8110196

**Published:** 2018-11-03

**Authors:** Tam T. Quach, Nathalie Auvergnon, Rajesh Khanna, Marie-Françoise Belin, Papachan E. Kolattukudy, Jérome Honnorat, Anne-Marie Duchemin

**Affiliations:** 1 Lyon Neuroscience Research Center INSERM U1028/CNRS UMR5292, F-69372 Lyon, France; quach.1@osu.edu (T.T.Q.); nathalie.auvergnon@univ-lyon2.fr (N.A.); marie-francoise.belin@lyon.inserm.fr (M.-F.B.);jerome.honnorat@chu-lyon.fr (J.H.); 2 Department of Neuroscience, The Ohio State University, Columbus, OH 43210, USA; 3 Departments of Pharmacology, University of Arizona, Tucson, AZ 85724, USA; rkhanna@email.arizona.edu; 4 The Center for Innovation in Brain Sciences, The University of Arizona Health Sciences, Tucson, Arizona 85724, USA; 5 Department of Biological Chemistry and Pharmacology, The Ohio State University, Columbus, OH 43210, USA; pk@ucf.edu; 6 Institut NeuroMyoGene Inserm U1217/CNRS UMR 5310, Université Claude Bernard Lyon1, Lyon, and Hospices Civils de Lyon, Hôpital Neurologique, Neurologie B, F-69677 Bron, France; 7 Department of Psychiatry, The Ohio State University, Columbus, OH 43210, USA

**Keywords:** dentate gyrus development, dentate granule cells, morphogenesis, CA3, dendrite, mossy fiber, CRMP3

## Abstract

Collapsin response mediator proteins (CRMPs) are highly expressed in the brain during early postnatal development and continue to be present in specific regions into adulthood, especially in areas with extensive neuronal plasticity including the hippocampus. They are found in the axons and dendrites of neurons wherein they contribute to specific signaling mechanisms involved in the regulation of axonal and dendritic development/maintenance. We previously identified CRMP3’s role on the morphology of hippocampal CA1 pyramidal dendrites and hippocampus-dependent functions. Our focus here was to further analyze its role in the dentate gyrus where it is highly expressed during development and in adults. On the basis of our new findings, it appears that CRMP3 has critical roles both in axonal and dendritic morphogenesis of dentate granular neurons. In CRMP3-deficient mice, the dendrites become dystrophic while the infrapyramidal bundle of the mossy fiber shows aberrant extension into the stratum oriens of CA3. This axonal misguided projection of granular neurons suggests that the mossy fiber-CA3 synaptic transmission, important for the evoked propagation of the activity of the hippocampal trisynaptic circuitry, may be altered, whereas the dystrophic dendrites may impair the dynamic interactions with the entorhinal cortex, both expected to affect hippocampal function.

## 1. Introduction

The dentate gyrus (DG) is involved in the pathophysiology of epilepsy, acute and chronic stress, mood disorders, and age-related cognitive dysfunction [1,2,3,4]. In mice, the DG has three layers [5]: (1) the molecular layer/stratum moleculare contains the dendrites of granule neurons (GN) as well as fibers of the perforant path that originate from the entorhinal cortex, and various inter-neurons types; (2) the granular layer/stratum granulosum consists of packed GNs. Their unmyelinated axons, the mossy fibers (MF), have two bundles: a short infrapyramidal bundle (IPB) and a long main bundle (MB), also referred to as stratum lucidum, which invades the hilar region, passing along the apical dendrites of CA3 pyramidal cells and collaterizing with basket cells before entering the CA3 field and contacting the proximal parts of the basal dendrites; (3) the hilar layer/hilus contains polymorphic cells [6]. 

During development, the wiring of the nervous system depends on the precise refinement of axons and dendrites arbors. The dendritic and axonal outgrowth—and later the retraction and pruning of their inappropriate branches—are important for neuronal circuitry development and brain activity/information processing [7,8,9]. Although several factors contributing to the development and maturation of dendrites and axons of DG have been identified [10,11,12], the understanding of the mechanisms regulating this highly organized neuroanatomical structure remains incomplete. Members of the collapsin response mediator protein (CRMP) family have been found to be critical as intermediate signaling molecules of neurotrophic/collapsing factors and guidance cues for the determination of axons and dendrite morphology in several areas of the brain [13,14]. In the present study, we show that the intracellular CRMP3 is required for proper axon-dendrite development of GNs, critical for the normal function of DG: deleting the *Crmp3* gene in vivo leads to a phenotype of decreased dendrite outgrowth but enhanced axon extension.

## 2. Materials and Methods

### 2.1. Mouse Breeding and Genotyping

All experiments were conducted in accordance with the guidelines of the National Institute of Health, USA. The CRMP3^−/−^ mouse line and polymerase chain reaction (PCR) genotyping were previously described [15]. At least 4–6 male mice CRMP3^−/−^ and wild-type (WT) littermates were used per condition. 

### 2.2. Golgi and X-gal Staining

For β-galactosidase staining, serially cut 30-µm-thick cryosections of fixed brains were incubated with X-gal solution (5 mM potassium ferricyanide, 5 mM potassium ferrocyanide, 2 mM MgCl_2_, and 1 mg/mL 5-bromo-4-chloro-3-indoyl-β-d-galactopyranoside in phosphate-buffered saline (PBS)) at 37 °C for 10–12 h. Golgi staining was performed according to the manufacturer’s instructions (Rapid Golgi Staining Kit, FD Neurotechnologies, Inc. Ellicott City, MD, USA). Coronal sections (150 µm) containing identical regions of the hippocampal formation were selected from WT and CRMP3^−/−^ mice for analysis. Neurons chosen for camera lucida tracing were entirely impregnated with Golgi stain, were not obscured by other neurons and all neurites were visible within the plane of focus. For quantification of the undulation of apical dendrites, a linearity index was calculated by computer-assisted measurement of apical dendrite lengths (100 µm from the cell body) using MCID Elite image analysis software (Imaging Research, Inc. St. Catherine’s, ON, Canada). The linearity index is defined as the curvilinear length in microns of a region of the apical dendrite divided by the linear distance between the ends of the region measured [16]. For spine morphology, spines were classified based on the category that most resembled the shape of that spine. The length of each spine was defined as the distance from the distal surface of the spine head to the dendrite in µm. For spine density, defined as number of spines per 25 µm of dendrite, spines were counted at 50 µm long distance from the soma in the stratum moleculare. Slides were coded prior to quantitative analysis by a blind-rater. 

### 2.3. Timm Staining

For Timm staining [17,18], sections were stained with a freshly prepared solution of 1.2 mM gum arabic, 0.15 M hydroquinone, and 0.05 M silver nitrate in sodium citrate buffer, fixed in photofixative then counterstained with Neutral Red.

### 2.4. Immunohistochemistry

Cryostat sections (10–20 µm) were collected on Superfrost Plus slides, permeabilized with 0.1% Triton X-100 in PBS containing 1% gelatin, and stained with the following antibodies: mouse monoclonal against MAP2 (Chemicon International, Temecula, CA, USA), rabbit polyclonal against MAP2 (Sigma, St. Louis, MO, USA), β-galactosidase ( Promega Madison, WI, USA), anti-neuropilin 1 (NP1; Abcam, Cambridge, MA, USA) and anti-neuropilin 2 (NP2; Sigma, St. Louis, MO, USA), neurofilament 200 (Biorad, Hercules, CA, USA), or anti-calbindin (Santa Cruz, Santa Cruz, CA, USA, ) antibodies. Sections were incubated with one or more of the secondary antibodies (Alexa Fluor 546-coupled anti-rabbit IgG and Alexa Fluor 488-coupled anti-mouse IgG; 1/2000, Molecular Probes, Eugene, OR, USA). Some sections were incubated with a 0.1 µg/mL solution of DAPI (4,6-diamidino-2-phenylindoldihydrochloride, Sigma) to label cell nuclei. Sections were viewed using an epifluorescent Zeiss microscope as previously described [19]. 

### 2.5. Neurite and Infrapyramidal Bundle (IPB) Length Quantification

Quantification of the IPB length was performed using the ratio of IPB length to the length of the CA3 as described by Bagri et al. [20]. 

### 2.6. Statistics

Quantitative data were expressed as mean ± standard error of the mean (SEM). The difference between two groups was calculated with an unpaired two-tailed Student *t* test. Statistical analysis was done with GraphPad-InSTat Version 3 software (La Jolla, CA, USA) with significance set at *p* < 0.05. 

## 3. Results

### 3.1. Collapsin Response Mediator Protein 3 (CRMP3)^−/−^ Dentate Gyrus

**Figure 1:**
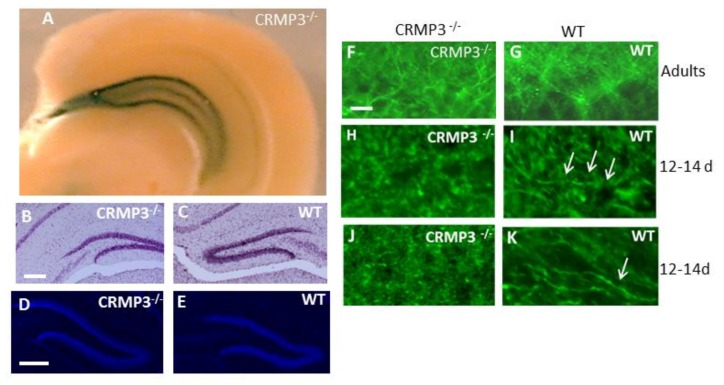
Collapsin response mediator protein 3 (CRMP3^−/−^) dentate gyrus characteristics. Coronal section of CRMP3^−/−^ adult brain shows *lacZ* activity driven by the *Crmp3* gene promoter in the hippocampus proper and the dentate gyrus (DG) (**A**). No gross abnormality was seen in DG of 3-month old CRMP3^−/−^ mice by light microscopy of cresyl violet (**B**,**C**), or DAPI (**D**,**E**)-stained sections. MAP2 staining revealed cytoarchitectural disorganization in the DG molecular layer of CRMP3^−/−^ (**F**) as compared to WT (**G**) in adults. Interestingly, there is a clear difference between the 2 genotypes of DG in 12–14 days old animals as visualized with MAP2 (**H**,**I**) and neurofilament 200 (**J**,**K**) staining. Scale bar: 200 µm in (**B**–**E**); 15µm in (**F**,**K**). White arrows in I and K point to well defined neurites present in WT and absent in CRMP3^−/−^.

The vector targeting the disruption of the *Crmp3* gene contains the *LacZ* gene that allows identification of CRMP3-expressing cells through visualization of β-galactosidase by immunohistochemistry or enzymatic assay. A dominant β-galactosidase distribution was found in hippocampus and especially in GN (Figure 1A, Appendix A) of CRMP3^−/−^ mice, confirming previous CRMP3 in situ hybridization data of the high distribution of CRPM3 in DG [21]. Although no gross DG anatomical abnormalities in adult CRMP3^−/−^ mice were observed by light microscopy of cresyl violet- (Figure 1B,C) or DAPI- (Figure 1D,E) stained sections of the hippocampus, there was a poor alignment and tortuous appearance of the dendritic arbor in DG from CRMP3^−/−^ mice (Figure 1F) visualized by MAP2 staining. In addition, a delay of neuritogenesis of GN was detected in 12- to 14-day-old CRMP3^−/−^ mice by MAP2 (Figure 1H,I) and neurofilament 200 (Figure 1J,K) staining.

### 3.2. Dystrophy of Dendrites in CRMP3^−/−^ Granular Neurons

**Figure 2:**
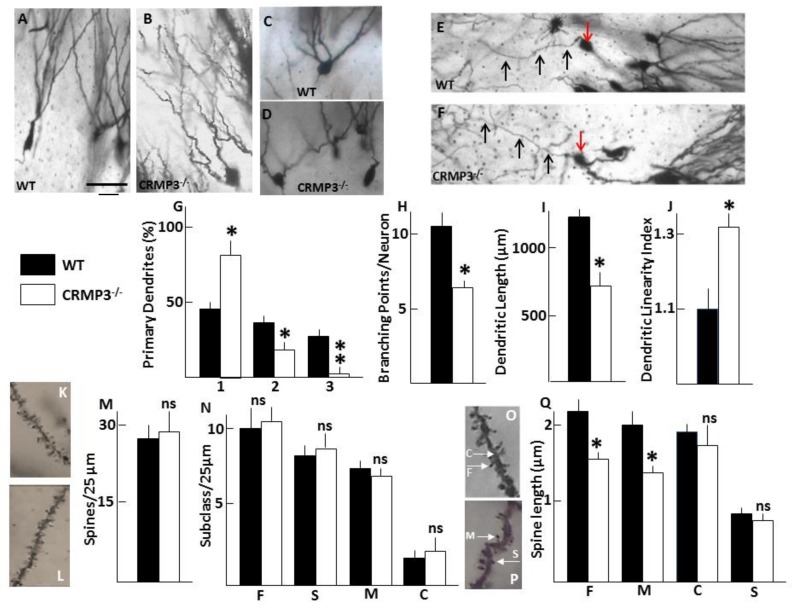
Morphometric analysis of dendrites and spines of granular neurons in adult CRMP3^−/−^ mice. *Morphometric analysis of dendrites*. A–D: Representative Golgi-stained granule neurons (GNs) and their proximal dendrites in wild-type (WT) (**A**,**C**) and CRMP3^−/−^ mice (**B**,**D**). (**E**,**F**): Representative axons (black arrows) from WT (**E**) and CRMP3^−/−^ (**F**) granular neurons (red arrows). PD: primary dendrite; SD: Secondary dendrite extending from PD; TD: Tertiary dendrite extending from SD. (**G**): Number of PDs/cell expressed as percentage of total cell count (WT, *n* = 253; CRMP3^−/−^, *n* = 275). (**H**): Number of branching points per neuron. (**I**): Average total dendritic length (PD + SD + TD) of 50 neurons per genotype. (**J**): dendritic linearity index from WT and CRMP3^−/−^ mice (*n* = 62 and 76, respectively). Scale bar: 50 µm in **A**–**F**. *Morphometric analysis of dendritic spines.* (**K**,**L**,**O**,**P**): Representative Golgi-stained spine density from WT (**K**) and CRMP3^−/−^ (**L**) mice and sub-types morphology (**O**–**P**; C = cup; M = mushroom; S = stubby; F = finger). (**M**): Graph illustrating the density of spines along 25 microns length (*n* = 120–135 measurements/genotype). (**N**): Graph illustrating the density of individual spines subtypes along 25 microns length (*n* = 480–500 measurements/genotype). (**Q**): Quantification of the length of the spines (*n* = 2000–2200 spines/genotype). The length of F and M spines is significantly different between genotypes. All statistics for quantitative analysis were performed using average measurement per animal with 4 animals for each genotype. Statistical significance was assessed by two-tailed *t* test, * *p* < 0.05; ** *p* < 0.01. Error bars indicate standard error of the mean (SEM).

To identify and quantify abnormalities of the dendrites present in adults, hippocampi were stained with the Golgi impregnation method. Data confirmed the presence of dendrite abnormalities associated with the absence of CRMP3 (Figure 2B,D) compared with WT (Figure 2A,C). First, the pattern of distribution of granular cells harboring 1, 2 or 3 primary dendrites (PDs) was different between the two genotypes: CRMP3^−/−^ mice had a significantly greater proportion of cells across the stratum moleculare with single PD, while WT mice had more cells with multiple PDs (Figure 2C,D,G). Second, the arborization pattern of the dendrites was changed. Quantitative analyses revealed a 40% reduction in total dendritic length of GNs in CRMP3^−/−^ mice (WT, 1316 ± 75 μm; CRMP3^−/−^, 772 ± 31 μm; Figure 2I), which was due mainly to the significant decrease in dendritic branching (Figure 2H). CRMP3^−/−^ GNs appeared to have fewer primary dendrites and less branching, resulting in a decrease in total dendritic length. Finally, GNs displayed more tortuous/undulating dendrites in CRMP3^−/−^ mice (Figure 2B) than in WT mice (Figure 2A). The mean linearity index [15] for dendrites of GNs from WT mice was 1.10 ± 0.03, whereas that of dendrites from CRMP3^−/−^ mice was 1.30 ± 0.02 (Figure 2J). The tortuous morphology was limited to dendrites and did not affect the axons of the same neurons (Figure 2E,F). 

### 3.3. Dendritic Spine Alterations in CRMP3^−/−^ Granular Neurons

Dendritic spines develop as small protrusions from the dendritic shaft, and changes in their shape and length influence Ca^2+^ fluxes and impair electrical signaling. As shown in Figure 2O,P, four subtypes of spines—stubby, finger, cup, and mushroom—were observed by Golgi staining of GNs in the DG of adult CRMP3^−/−^ mice and there was no significant difference in total spine density (Figure 2K–M) or in density of individual subtypes of spines (Figure 2N) between genotypes. However, mushroom and finger spines in the stratum moleculare were significantly shorter in CRMP3^−/−^ than in WT mice (Figure 2Q). 

### 3.4. Mossy Fiber Outgrowth in CRMP3^−/−^ Mice

**Figure 3:**
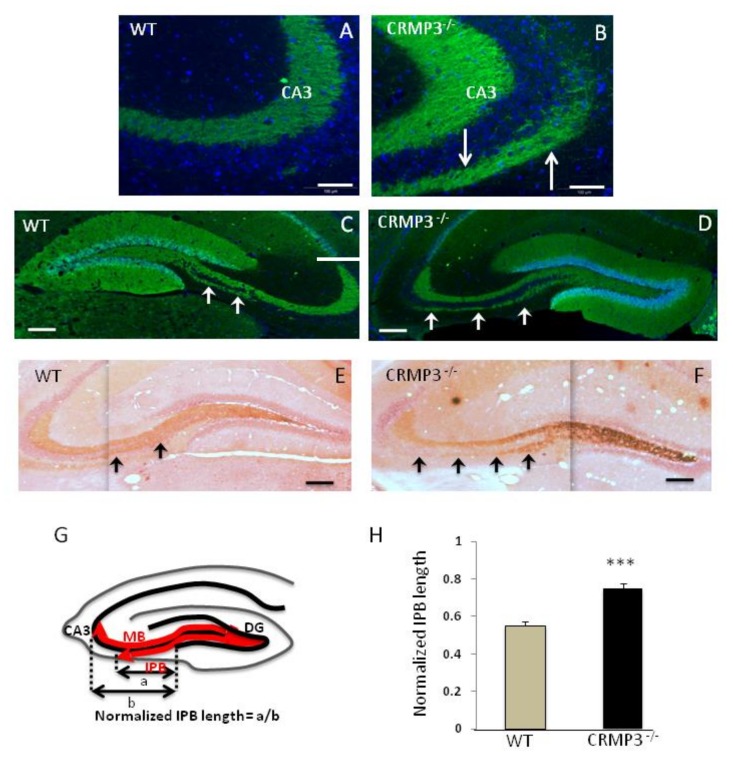
Altered infrapyramidal bundle (IPB) in CRMP3^−/−^ mice. (**A**–**F**): Free-floating coronal (**A**,**B**) and sagittal (**C**–**F**) hippocampal sections (20µm) from 2.5 month old mice (*n* = 5 for each genotype) immunostained with antibodies to calbindin (green) and DAPI (blue) (**A**–**D**) or with sulfide silver (**E**,**F**) from WT (**A**,**C**,**E**) or CRMP3^−/−^ (**B**,**D**,**F**) mice. In WT, the IPB stops before the beginning of the CA3 curvature (**A**) white arrows; and (**C**) black arrows, whereas it extends clearly beyond it in CRMP3^−/−^ mice (**D**), white arrows; and (**F**) black arrows. (**G**) Schematic drawing of mossy fibers (MF) showing their dentate origin and their characteristic trajectories (MB: main bundle). (**H**): Quantitation and analyses of IPB length. The IPB length “a” was measured from the tip of the inferior blade of the granular layer and the length of CA3, “b” was determined from the tip of the inferior blade to the apex of the curvature of the CA3 pyramidal cell layer. Quantitation of normalized IPBs defined as a/b confirms the abnormal IPB in the mutants. Statistical significance was assessed by two-tailed *t* test, *** *p* < 0.01. Scale bar = 50µm in (**A**,**B**); 200µm in (**C**,**F**). Error bars indicate standard error of the mean (SEM).

In adult WT, MF, which give rise to the main long MB and the short thin IPB, can be visualized by several techniques, including calbindin immunohistochemical staining [22] and Timm sulfide silver staining [17]. Surprisingly, we found an intriguing pattern of calbindin-immunostaining in the hippocampi of CRMP3^−/−^ mice (Figure 3B, white arrows), absent in the corresponding regions in WT mice (Figure 3A). We visualized the two bundles on sagittal sections and examined the extent of their growth that confirmed the striking phenotype difference of the IPB between the two genotypes. In WT adult mice, the IPB is short and the axons do not extend to the apex of the curvature of CA3 (Figure 3C white arrows, Figure 3E black arrows). In contrast, in CRMP3^−/−^ mice, there is an intense Timm labeling in the hilar area (Figure 3F, white arrow) and longer extensions of the IPB up to the CA3 excrescence (Figure 3D white arrows, Figure 3F black arrows). The normalized IPB length displayed a significant (*p* < 0.01) 36% increase in CRMP3^−/−^ compared to WT (Figure 3G–H).

## 4. Discussion

Different members of the CRMP family play critical signaling roles in axon/dendrite development [13,23]. Previously, we cloned the gene encoding CRMP3 [21] and by in vivo targeting the gene showed that CRMP3-deficient mice have dystrophic dendrites in the area CA1 of the hippocampus [15]. In vitro experiments have confirmed the role of CRMP3 in the regulation of dendrite morphology: cultured CRMP3^−/−^ hippocampal pyramidal neurons were characterized by the absence of neuritogenesis while overexpression of CRMP3 induced lamellipodia formation, neurite initiation, and dendritic outgrowth via activation of voltage-gated Ca^2+^ channels [19]. In addition, CRMP3 conferred protection to dendrites against dystrophy induced by the prion peptide PrP106-126 [24]. Here, we found that dendrites from CRMP3^−/−^ dentate granular neurons displayed abnormalities similar to those seen in CA1, including a delay in dendritogenesis and a decrease in dendritic length and branching points associated with an excessive undulation index. This excessive undulation is likely due to the altered signaling of CRMP3-dependent guidepost proteins or intermediate cues/targets directing point-to-point navigation of the dendrite growth cone [25,26]. The positive and negative influences of guidepost proteins [27] may not be transduced into the correct steering mechanisms in the CRMP3^−/−^ growth cones resulting in a saltatory behavior and an undulated morphology of dendrites. Similar alterations are also recapitulated in mice deficient for the cell adhesion molecule L1, which is a co-receptor of the neuropilin NP1/Sema3A receptor complex [16,28]. Moreover, CRMP3^−/−^ GN appeared to have fewer primary dendrites, less branching and a decrease in total dendritic length. These present (Figure 2A–J) and previous observations [15] together with our hippocampal micro-array gene expression profiling (showing a more than 40% decrease in MAP2 and MAP5 expression (Appendix B), strongly argue in favor of a critical role of CRMP3 as an intrinsic molecule required for dendrite morphogenesis in hippocampus. Furthermore, we also observed changes in the size of the mushroom and finger spines (Figure 2K–Q) which have been implicated in memory and learning, respectively [29].

Axons differ profoundly from dendrites in their morphology and subcellular organization (i.e., microtubules orientation)/composition (i.e., Tau1/MAP2). From this perspective, we showed that mutation of an intracytoplasmic protein, CRMP3, induces opposing changes in the overall morphology of axons versus dendrites: in the same neuron type, dendritic dystrophy contrasts to axonal extension. To be precise, regarding the axons of GN, whereas the WT IPB extended only below the pyramidal layer, the CRMP3^−/−^ IPB stretched out to the longitudinal septotemporal curve of CA3. Interestingly, a similar mis-pathfinding phenotype was observed with the gene targeting studies of Sema 3F [30] and its receptors complex NP2 [31] and PLXA3 [32]: the IPB of these mutant mice extends throughout the CA3 excrescence during development, and is not pruned back between postnatal days 20 to 30 as compared to WT mice. The pruning involves a central role of Sema 3F (acting through a neuropilin 2/plexin 3A receptor complex [20]). In contrast, the micro-infusion of neurotrophins in the brain induces dendrites [33] and MF [34] outgrowth. Interestingly, in CRMP3^−/−^ mice, subsets of LacZ^+^ cells in DG were immunoreactive for guidance cue receptors (including NP1 and NP2 (Appendix C) and TrKs (not shown). Whether CRMP3 is involved in the signaling pathways of specific extra-cellular guidance cues, i.e. Sema 3F, neurotrophins and/or other molecules in dentate GNs remains to be elucidated. The abnormal neurite structures present in the hippocampus of CRMP3-deficient mice may explain the altered hippocampal functions we observed in these animals, including long-term potentiation elicited by the application of theta burst stimulation [15] and prepulse inhibition [35]. 

## 5. Conclusions

Our present findings from genetic manipulation suggest that CRMP3 has critical roles both in the axon and dendrite morphogenesis of neurons in DG and CA1, and position the protein as an important intracytoplasmic determinant of hippocampal neuronal structure and, consequently a modulator of the hippocampal trisynaptic circuitry function implicated in chronic stress and depression [36], schizophrenia [37], age-associated neurodegeneration, and memory loss [38].

## Figures and Tables

**Figure A1:**
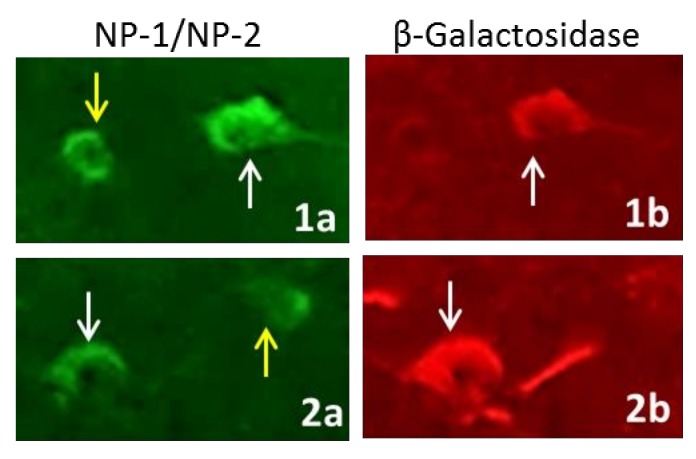
Immunohistochemistry for guidance cue receptors in CRMP3^−/−^ mice. Co-localization of NP-1 (**1a**) and NP-2 (**2a**) with β-galactosidase (**1b**,**2b**) in CRMP3^−/−^ DG. White arrows point at cells positive both for NP-1^+^ and β-galactosidase^+^ (**1a** and **1b**) or both NP-2^+^ and β-galactosidase^+^ (**2a** and **2b**). Yellow arrows point to cells positive for NP-1^+^ (**1a**) or NP-2^+^ (**2a**) and negative for β-galactosidase^−^. Scale bar: 10µm in 2a-3b.

## Appendix A

**Table A1:** Regional distribution of CRMP3 in the nervous system of post-natal mice. LacZ activity, as index of CRMP3 expression, was strongest in DG (ND: not detectable, *: present in one week old animals).

CRMP3 expression in 5-week old mice	
CA1; CA3	+++
Dentate Gyrus	+++++
Cerebellum (Granular Cells)	+
Thalamus	++
Hypothalamus	+
Pons (Olivary Complex)	+++
Reticular Formation	+
Cortex, Striatum	ND
Spinal Cord	ND/*
Dorsal Root Ganglia	ND/*

## Appendix B

**Table A2:** Hippocampal DNA micro-array. Complementary DNA was reversed transcribed from the mRNA extracted from WT and CRMP3^−/−^ hippocampi (3 months old; 8 animals/genotype), and then hybridized to microarrays. Following hybridization, the arrays were washed and scanned following standard Affymetrix protocols (Affymetrix GeneChip Genome arrays; Affymetrix, Santa Cruz, CA, USA). Genes with change in mRNA expression of more than 30% are listed.

Accession Number	Gene Description	Direction of Change (%)	
		Decrease	Increase
M86567	GABA-A receptor, subunit alpha2	−32	
M64068	Zinc Finger Protein (bmi-1)	−34	
AF033003	K+ channel beta-1 subunit (KVB1)	−36	
M37335	Myelin proteolipid protein	−38	
HO5479	Calcineurin catalytic subunit	−39	
M21041	MAP2	−41	
X51396	Map5	−42	
U34973	Protein tyrosine phosphatase-like	−46	
AB022342	Glutamate receptor channel, subunit α3	−51	
U42384	FGF inducible 15	−56	
X57498	Glutamate receptor AMPA2, subunit α2	−70	
X61430	GABA-A receptor, subunit α1	−72	
X84234	Ganglioside GM 1		+152
AF037437	Prosaposin		+166
X76858	E4F transcription factor		+218
X95518	neuronal tyrosine/threonine phosphatase 1		+226
X72305	Corticotropin releasing hormone receptor		+248

## References

[1] Lee J.W., Jung M.W. (2017). Separation or binding? Role of the dentate gyrus in hippocampal mnemonic processing. Neurosci. Biobehav. Rev..

[2] Nakahara S., Adachi M., Ito H., Matsumoto M., Tajinda K., Van Erp T.G.M. (2018). Hippocampal Pathophysiology: Commonality Shared by Temporal Lobe Epilepsy and Psychiatric Disorders. Neurosci. J..

[3] Cameron H.A., Schoenfeld T.J. (2018). Behavioral and structural adaptations to stress. Front. Neuroendocrinol..

[4] Kesner R.P. (2018). An analysis of dentate gyrus function (an update). Behav. Brain Res..

[5] Schultz C., Engelhardt M. (2014). Anatomy of the hippocampal formation. Front. Neurol. Neurosci..

[6] Lauder J.M., Mugnaini E. (1980). Infrapyramidal mossy fibers in the hippocampus of the hyperthyroid rat. A light and electron microscopy study. Dev. Neurosci..

[7] Riccomagno M.M., Kolodkin A.L. (2015). Sculpting neural circuits by axon and dendrite pruning. Annu. Rev. Cell Dev. Biol..

[8] Schuldiner O., Yaron A. (2015). Mechanisms of developmental neurite pruning. Cell. Mol. Life Sci..

[9] Ledda F., Paratcha G. (2017). Mechanisms regulating dendritic arbor patterning. Cell. Mol. Life Sci..

[10] Puram S.V., Bonni A. (2013). Cell-intrinsic drivers of dendrite morphogenesis. Development.

[11] Copf T. (2015). Importance of gene dosage in controlling dendritic arbor formation during development. Eur. J. Neurosci..

[12] Koyama R., Ikegaya Y. (2018). The Molecular and Cellular Mechanisms of Axon Guidance in Mossy Fiber Sprouting. Front. Neurol..

[13] Quach T.T., Lerch J.K., Honnorat J., Khanna R., Duchemin A.M. (2016). Neuronal networks in mental diseases and neuropathic pain: Beyond brain derived neurotrophic factor and collapsin response mediator proteins. World J. Psychiatry.

[14] Nagai J., Baba R., Ohshima T. (2017). CRMPs Function in Neurons and Glial Cells: Potential Therapeutic Targets for Neurodegenerative Diseases and CNS Injury. Mol. Neurobiol..

[15] Quach T.T., Massicote G., Belin M.F., Honnorat J., Glasper E.R., Devries A.C., Jakeman L., Baudry M., Duchemin A.M., Kolattukudy P.E. (2008). CRMP3 is required for hippocampal CA1 dendritic organization and plasticity. FASEB J..

[16] Demyanenko G.K., Tsai A.Y., Maness P.F. (1999). Abnormalities in neuronal process extension, hippocampal development, and the ventricular system of L1 knockout mice. J. Neurosci..

[17] Timm F. (1958). Zur histochemie der Schwermetalle. Das Sulfid-Silber-verfahren. Disch. Z. Ges. Gerichtl. Med..

[18] Slomianka L. (1992). Neurons of origin of zinc-containing pathways and the distribution of zinc-containing bouton in the hippocampal region of the rat. Neuroscience.

[19] Quach T.T., Wilson S.M., Rogemond V., Chounlamountri N., Kolattukudy P.E., Martinez S., Khanna M., Belin M.F., Khanna R., Honnorat J. (2013). Mapping CRMP3 domains involved in dendrite morphogenesis and voltage-gated calcium channel regulation. J. Cell Sci..

[20] Bagri A., Cheng H.J., Yaron A., Pleasure S.J., Tessier-Lavigne M. (2003). Stereotyped pruning of long hippocampal axon branches by retraction inducers of the semaphorin family. Cell.

[21] Quach T.T., Mosinger B., Ricard D., Copeland N.C., Gilbert D.J., Jenkins J.A., Stankford B., Honnorat J., Belin M.F., Kolattukudy P.E. (2000). Collapsin response mediator protein 3/unc33-like protein 4 gene: Organization, chromosomal mapping and expression in the developing mouse brain. Gene.

[22] Sloviter R.S. (1989). Calbindin and parvalbumin immunocytolocalization in the rat hippocampus with specific reference to the specific vulnerability of hippocampal neurons to seizure activity. J. Comp. Neurol..

[23] Quach T.T., Honnorat J., Kolattukudy P.E., Khanna R., Duchemin A.M. (2015). CRMPs: Critical molecules for neurite morphogenesis and neuropsychiatric diseases. Mol. Psychiatry.

[24] Quach T.T., Wang Y., Khanna R., Chounlamountri N., Auvergnon N., Honnorat J., Duchemin A.M. (2011). Effect of CRMP3 expression on dystrophic dendrites of hippocampal neurons. Mol. Psychiatry.

[25] Bentley D., Caudy M. (1983). Pioneer axons lose directed growth after selective killing of guidepost cells. Nature.

[26] O’Connor T.P., Bentley J.S. (1990). Accumulation of actin in subsets of pioneer growth cone filopodia in response to neural and epithelial guidance cues in situ. J. Cell. Biol..

[27] Kuhn T.B., Schmidt M.F., Kater S.B. (1995). Laminin and fibronectin guideposts signal sustained but opposite effects to passing growth cones. Neuron.

[28] Bechara A., Nawabi F., Moret F., Yaron A., Weaver E., Bozon M., Abouzid K., Guan J.L., Tessier-Lavigne M., Lemmon V. (2008). FAK-MAPK-dependent adhesion disassembly downstream of L1 contributes to semaphorin 3A-induced collapse. EMBO J..

[29] Berry K.P., Nedivi E. (2017). Spine Dynamics: Are They All the Same?. Neuron.

[30] Sahay A., Molliver M.E., Ginty D.D., Kolodkin A.L. (2003). Semaphorin 3F is critical for development of limbic system circuitry and is required in neurons for selective CNS axon guidance events. J. Neurosci..

[31] Chen H., Bagri A., Zupicich J.A., Zou Y., Stoeckli E., Pleasure S.J., Lowenstein D.H., Skarmes W.C., Chedotal A., Tessier-Lavigne M. (2000). Neuropilin-2 regulates the development of select cranial and sensory nerves and hippocampal mossy fiber projections. Neuron.

[32] Cheng H., Bagri A., Yaron A., Stein E., Pleasure S.J., Tessier-Lavigne M. (2001). Plexin-3A mediates semaphorin signaling and regulates the development of hippocampal axonal projection. Neuron.

[33] Li M., Dai F.R., Du X.P., Yang Q.D., Zhang X., Chen Y. (2012). Infusion of BDNF into the nucleus accumbens of aged rats improves cognition and structural synaptic plasticity through PI3K-ILK-AKt signaling. Behav. Brain Res..

[34] Ramos-Languren L.E., Escobar M.L. (2013). Plasticity and metaplasticity of adult rat hippocampus mossy fibers induced by neurotrophin-3. Eur. J. Neurosci..

[35] Quach T.T., Glasper E.R., Devries C., Honnorat J., Kolattukudy P.E., Duchemin A.M. (2008). Altered prepulse inhibition in mice with dendrite abnormalities of hippocampal neurons. Mol. Psychiatry..

[36] Stepan J., Hladky F., Uribe A., Holsboer F., Schmidt M.V., Eder M. (2015). High-Speed imaging reveals opposing effects of chronic stress and antidepressants on neuronal activity propagation through the hippocampal trisynaptic circuit. Front. Neural Circuits.

[37] Benes F.M. (1999). Evidence for altered trisynaptic circuitry in schizophrenic hippocampus. Bio. Psychiatry..

[38] Moorthi P., Premkumar P., Priyanka R., Jayachandran K.S., Anusuyadevi M. (2015). Pathological changes in hippocampal neuronal circuits underlie age-associated neurodegeneration and memory loss: Positive clue toward SAD. Neuroscience.

